# Enhancing quality of antimicrobial prescribing through ‘Ask Eolas’ (language model): a user-testing and simulation evaluation

**DOI:** 10.1038/s44259-026-00187-7

**Published:** 2026-03-03

**Authors:** William J. Waldock, Mark Gilchrist, Hutan Ashrafian, Ara Darzi, Bryony Dean Franklin

**Affiliations:** 1 Institute of Global Health Innovation, Imperial College London, London, UK; 2 Imperial College Healthcare NHS Trust, London, UK; 3 NIHR Northwest London Patient Safety Research Collaboration, London, UK; 4 UCL School of Pharmacy, London, UK

**Keywords:** Health care, Medical research

## Abstract

We aimed to assess prescribing accuracy, error reduction, usability, and clinician confidence of Ask Eolas (a retrieval-augmented generation-enhanced AI-CDSS) compared to existing antimicrobial guidance tools. We conducted a structured simulation single-site study evaluating Ask Eolas across 45 prescribing cases with healthcare professionals to assess prescribing accuracy. Among 45 participants, Ask Eolas achieved zero prescribing errors versus six and eight documented errors in the two comparator groups (Eolas App and PDF Guidelines), respectively (*p* < 0.001). The number needed to treat was 1.9 for Ask Eolas versus traditional guidelines, indicating one additional error-free prescription for every two clinicians switching to Ask Eolas. Ask Eolas significantly improved prescribing accuracy while enhancing usability, clinician confidence, and system transparency compared to existing tools. These findings align with TRUST-AI framework principles for safe AI-CDSS deployment, supporting further investigation through real-world implementation studies incorporating live data integration, confidence calibration systems, and comprehensive auditability features in antimicrobial stewardship programmes.

## Introduction

The escalating crisis of antimicrobial resistance (AMR) poses a critical global health threat, driven by inaccurate prescribing and the widespread use of broad-spectrum antibiotics^1^. Clinical decision support systems (CDSS) are integral to antimicrobial stewardship^2^, offering guidance to improve prescribing decisions. However, widespread adoption remains constrained by usability limitations, lack of trust, and integration barriers within clinical workflows^3^. Artificial intelligence (AI), particularly large language models (LLMs), has redefined the potential of clinical support tools. Yet, previous LLMs often lacked transparency, consistency, and domain alignment^4^; this requires aligning the CDSS with the clinical and electronic prescribing (EP) environment. Retrieval-augmented generation (RAG) addresses these issues by coupling LLMs with access to curated, evidence-based guidelines, thereby enhancing both factual reliability and clinical alignment^5^.

Refining LLMs for clinical decision-making requires a structured framework that emphasises domain-specific knowledge integration, rigorous validation, and real-world usability testing^6^. Employing decision-mapping frameworks helps define clear points where AI intervention can provide the greatest value^7^, while behavioural science insights inform strategies to enhance user engagement and confidence^8^. Recognising the nuances in antimicrobial prescribing across surgical and medical specialities further ensures that the recommendations remain relevant and actionable^9^. A combination of these approaches directly addresses common limitations of existing CDSS by enhancing factual accuracy and ensuring alignment with evolving clinical standards^6^. Moreover, by leveraging RAG, the system dynamically retrieves the most current evidence during the recommendation process, thereby supporting prescribers in navigating complex antimicrobial decisions.

‘Ask Eolas’ is an additional development feature within the established Eolas Medical App, which is used to access hospital-authored clinical guidelines in approximately 80% of hospitals in England; as a RAG-enhanced CDSS, it integrates hospital guidelines into a specific design to support complex decision-making in antimicrobial therapy and speciality-specific pathways. The system addresses longstanding CDSS limitations through guideline retrieval. This evaluation focuses on assessing prescription accuracy, with secondary evaluations of usability and clinician confidence, aiming to determine whether RAG-enhanced AI tools can effectively bridge gaps in antimicrobial stewardship and improve clinical decision-making^10^. Additionally, this evaluation acknowledges key challenges in the implementation of Retrieval-augmented Generation CDSS: successful adoption requires seamless integration with prescribers’ diagnostic and prescribing workflows, speciality-specific adaptation, and a strong emphasis on usability and clinician trust ^9,11^. This product is an “AI-driven CDSS” because the RAG mechanism, which uses a pre-trained LLM for semantic search and synthesis, performs a sophisticated function far beyond simple keyword retrieval. The tool is an intelligent system that processes user context, retrieves multiple relevant extracts from documents of interest, and generates a concise, synthesised output in natural language.

This study presents a structured, three-phase simulated user testing evaluation of Ask Eolas through simulation prescribing across case complexity tiers. Our aim was to assess the prescribing accuracy of Ask Eolas in comparison to Eolas and the traditional presentation of antimicrobial guidelines. Accurate prescribing is understood to be a prescription without error, according to the official hospital's empirical antibiotic prescribing guidelines. We further examined how usability design and confidence stratification impact clinician trust and workflow integration.

## Results

### Participant demographics

The study follows the DECIDE-AI reporting guidelines and complies with the Standardised Iterative Reporting and Outcomes Simulation (SIROS) framework to ensure rigorous evaluation of early-phase AI systems^12,13^. Three groups of 15 healthcare professionals each completed the study protocol across all three intervention arms (total *n* = 45, 100% completion rate). Participant demographics were well-balanced across groups, with professional roles distributed proportionally among consultants (*n* = 16), pharmacists (*n* = 9), speciality registrars (*n* = 7), foundation doctors (*n* = 6), core trainee doctors (*n* = 4), and prescribing nurses (*n* = 3) (Table 1).

**Table 1:** Participant demographics and accurate prescription rates by intervention group

Professional role	Trust guidelines (*n* = 15)	Eolas App (*n* = 15)	Ask Eolas (*n* = 15)	Total (*n* = 45)
Consultant	6	5	5	16
Specialty registrar	3	2	2	7
Pharmacist	3	3	3	9
Foundation doctor	2	2	2	6
Core trainee doctor	0	2	2	4
Prescribing nurse	1	1	1	3
**Accurate prescriptions**	**7/15 (47%)**	**9/15 (60%)**	**15/15 (100%)**	**31/45 (69%)**

### Primary outcomes

The Ask Eolas intervention demonstrated superior performance with perfectly accurate prescription rates (100%, 15/15), significantly outperforming both the Eolas App (60%, 9/15) and Trust Guidelines (47%, 7/15) approaches. The overall accurate prescription rate across all participants was 69% (31/45), with a clear hierarchy of effectiveness: Ask Eolas above Eolas App above Trust Guidelines (Table 1). Fisher’s exact test revealed statistically significant improvements in prescribing accuracy for Ask Eolas compared to both Trust Guidelines (*p* < 0.001) and the Eolas App (*p* < 0.001), with absolute risk reductions of 53% and 40%, respectively, per prescribing episode. The number needed to treat (NNT) for Ask Eolas versus Trust Guidelines was 1.9, indicating that for every 2 clinicians using Ask Eolas instead of Trust Guidelines, one additional accurate prescription would result per clinical case, demonstrating substantial clinical utility. The inaccuracies observed using the original Trust Guidelines were overwhelmingly related to incorrect duration and incorrect dose, often due to misinterpretation of complex flowcharts or nested text.

### Secondary outcomes

Ask Eolas demonstrated markedly superior user experience compared to both Trust Guidelines and Eolas App (Table 2), with participants reporting the highest prescribing confidence (median 94 vs 72 and 68) and dramatically reduced cognitive load across all NASA-TLX domains. Mental demand, time pressure, effort required, and frustration levels were consistently lower with Ask Eolas compared to the other interventions. The Ask Eolas system also achieved the highest perceived performance scores, indicating users felt more successful when using the interactive AI consultation tool.

**Table 2:** Prescribing confidence and cognitive load

Metric	Trust guidelines: median (IQR*)	Eolas App: median (IQR*)	Ask Eolas: median (IQR*)
**Prescribing confidence (0−100)**	72 (54–89)	68 (49–86)	94 (91–97)
**NASA-TLX domains (0−100)**			
Mental demand	37 (23–52)	36 (24–49)	14 (8–19)
Physical demand	30 (18–42)	26 (15–37)	10 (6–14)
Time pressure	39 (26–53)	37 (24–50)	8 (5–12)
Effort required	41 (28–55)	41 (27–55)	10 (7–14)
Perceived performance	66 (52–79)	64 (50–78)	86 (82–91)
Frustration level	40 (27–54)	38 (25–51)	8 (4–13)

*IQR* interquartile range, *NASA-TLX* National Aeronautics and Space Administration Task Load Index.

System usability scores (Table 3) consistently favoured Ask Eolas across all ten SUS components, with participants rating it highest for frequency of desired use (4.9), ease of use (4.9), and user confidence (4.8) while scoring lowest on negative attributes like complexity (1.0) and cumbersomeness (1.3). The Eolas App generally outperformed Trust Guidelines but remained substantially below Ask Eolas performance levels. Overall, Ask Eolas achieved superior usability ratings, suggesting the interactive AI interface provides a more intuitive and user-friendly experience than either traditional guidelines or static digital applications.

**Table 3:** System Usability Scale (SUS) Components

SUS item	Trust guidelines: median (IQR)	Eolas App: median (IQR)	Ask Eolas: median (IQR)
1. Would like to use frequently	3.5 (3.0–4.0)	3.7 (3.2–4.2)	4.9 (4.8–5.0)
2. Unnecessarily complex^a^	2.5 (2.0–3.0)	2.4 (1.9–2.9)	1.0 (1.0–1.5)
3. Easy to use	3.4 (2.9−3.9)	3.7 (3.2−4.2)	4.9 (4.8−5.0)
4. Need technical support^a^	2.3 (1.8−2.8)	1.6 (1.1−2.1)	1.1 (1.0−1.2)
5. Functions well integrated	3.2 (2.7−3.7)	3.8 (3.3−4.3)	4.6 (4.3−4.9)
6. Too much inconsistency^a^	2.4 (1.9−2.9)	1.7 (1.2−2.2)	1.3 (1.0−1.6)
7. People would learn quickly	3.3 (2.8−3.8)	3.8 (3.3−4.3)	4.8 (4.6−5.0)
8. Cumbersome to use^a^	2.6 (2.1−3.1)	2.2 (1.7−2.7)	1.3 (1.0−1.6)
9. Felt confident using	3.3 (2.8−3.8)	3.5 (3.0−4.0)	4.8 (4.6−5.0)
10. Needed to learn many things^a^	2.3 (1.8−2.8)	1.8 (1.3−2.3)	1.3 (1.0−1.6)

^a^Lower scores indicate better usability for negatively worded items.

Participants valued Ask Eolas’ direct links to source guidelines, with one clinician noting it “helped me trust the answer.” Ask Eolas’ ability to provide concise, targeted summaries significantly reduced the information processing burden. Users appreciated the “short, clear summary” format, contrasting with Trust Guidelines that “overloaded me with text… no filtering.” The natural language processing capabilities of Ask Eolas enabled personalised recommendations. As one participant observed: “Ask Eolas understood patient context and offered tailored suggestions…[I] felt confident and reassured.”. Trust Guidelines were criticised for being “time-consuming and hard to pinpoint guidance,” which was particularly problematic in high-complexity clinical cases. However, limitations identified with Ask Eolas included occasional system latency (“took a few seconds too long to load sometimes”) and preference mismatches regarding narrative format length. The NVivo analysis framework is presented in Supplementary Notes 3.

## Discussion

On initial simulation evaluation, the Ask Eolas tool demonstrated better performance than comparison with no prescribing errors recorded; this outperformed both the Eolas App at 60% (95% CI: 32.3−83.7%) and Trust Guidelines at 47% (95% CI: 21.3−72.8%), with Fisher’s exact test confirming significant between-group differences. The clinical significance becomes interesting due to the number needed to treat of 1.9 for Ask Eolas versus Trust Guidelines, indicating that for every two clinicians switching to Ask Eolas from Trust Guidelines, one additional accurate prescription would result, demonstrating the tool’s high efficiency in improving antimicrobial prescribing decisions among healthcare professionals.

Alternatively, the Retrieval Augmented Generation Assessment framework^14^ offers a contrasting evaluation approach for retrieval-augmented systems, which importantly differ from traditional clinical decision support assessment. Unlike the rule-based system evaluated by Feather et al.^15^ using established mixed-methods approaches, AI-powered systems require metrics encompassing faithfulness, retrieval quality, and calibration. The preservation of antibiotics as shared resources^16^ highlights broader theoretical challenges in antimicrobial stewardship research. Feather’s study employed NASA-TLX workload assessment^17^ and demonstrated substantial error reduction through conventional approaches, yet the economic principles underlying prescribing behaviour^18^ suggest that individual decisions may not align with optimal population outcomes. Moreover, Coase’s social cost framework^19^ provides a theoretical foundation for understanding how negative externalities from inaccurate prescribing are not internalised by individual prescribers. Ostrom’s governance frameworks for common pool resources^20^ suggest that successful interventions require institutional mechanisms aligning individual incentives with collective benefits. Market-based approaches, including advance purchase commitments^21^, offer complementary strategies for addressing economic misalignments in prescribing behaviour.

Meanwhile, the psychology of cooperation under uncertainty^22^ becomes critical for the implementation of this technology. The tragedy of the commons in antimicrobial chemotherapy^23^ creates scenarios relevant to game theory in which individual prescribers face incentives favouring prescribing despite collectively suboptimal outcomes. The 50-percentage point improvement with Ask Eolas compared to traditional guidelines signals potential performance gains in clinical decision support efficacy. Contemporary optimisation approaches for antimicrobial therapy^24^ emphasise comprehensive interventions addressing multiple factors simultaneously. Notably, the complexity of resistance development and the impact on drug discovery^25^ reinforce the urgency of prevention-focused strategies. National surveillance data^26^ provides epidemiological foundations for intervention prioritisation, while systematic review evidence demonstrates that hospital-based prescribing interventions can achieve significant improvements^27^. Professional stewardship guidelines^28^ emphasise integrating clinical decision support within broader organisational frameworks. The development of AI-centred accuracy reporting standards^29,49^ assists the progression of rigorous evaluation methodologies, enabling systematic comparison between AI-supported and conventional approaches in real-world clinical contexts.

These simulation findings signpost the possibility that tools such as Ask Eolas have the potential to support safer, more efficient, and guideline-concordant antimicrobial prescribing. Whilst only a simulation evaluation, the system’s explainable outputs are reported to help build clinician trust, an essential step toward adoption of AI in high-stakes decision-making. This evaluation provides early-phase evidence that large language model (LLM)-based CDSS, such as Ask Eolas, have the potential to enhance prescribing accuracy, strengthen clinician trust, and support safer antimicrobial decision-making^30–33^. Considering these findings, we propose the TRUST-AI framework, with each component below derived directly from empirical observations and system behaviours identified during the study. We explain the development of each component herein.

### Transparency and trustworthiness (T)

Prescribers rated Ask Eolas highly in terms of usability and reported increased confidence in decision-making, as demonstrated by improvements in pre- and post-intervention confidence scores^31,34,37^. Participants regularly engaged with transparency features, including source citations, and the display of clinical rationale^30,32^. These findings underscore the central role of explainability in fostering trust in AI-CDSS^33,34^. Unlike earlier systems criticised for their opacity or the risk of clinician over-reliance^42,43^, Ask Eolas incorporated structured transparency mechanisms that helped mitigate these concerns, such as guideline citations. As a policy implication, transparency features such as direct citations of clinical guidelines should be mandated in regulatory submissions for AI-CDSS^45,47^. Further research is also needed to explore how interface design influences explainability, trust calibration, and clinician willingness to override AI recommendations^47^. While the current RAG implementation provides source citations, it would be improved by focusing on Chain-of-Thought reasoning to better display the clinical reasoning pathway.

### Real-time data integration (R)

While the current implementation of Ask Eolas used structured simulations, it did not incorporate live microbiology or pharmacology inputs^35,36^. Although the tool performed well under simulated conditions^30,38^, a deployment utilising the system’s full technical potential will necessitate richer, real-time data streams to further personalise treatment recommendations^39,40^. Policy should prioritise funding for the development of AI-CDSS pipelines capable of integrating real-time laboratory results, pharmacokinetic/pharmacodynamic (PK/PD) parameters, and antimicrobial resistance surveillance data^36,41^. Additionally, adherence to interoperability standards will be helpful for integration into existing EHR systems^39^. Integrating clinical data from the EHR is the next critical phase for this system, allowing for personalised, real-time decision support beyond guideline retrieval.

### Usability and user-centred design (U)

Usability emerged as a key strength of the system^31,37^. SUS scores were consistently high, and NASA-TLX assessments indicated low cognitive workload^33,37^. Think-aloud sessions revealed that participants intuitively grasped the logic of the interface and understood how to interpret confidence cues^30,32^. These results highlight the importance of human-centred design in the development of AI-CDSS tools^45,46^. Usability directly influenced decision confidence, workflow efficiency, and prescribing accuracy^37,47^. Regulatory bodies and procurement processes should mandate compliance with usability standards^46^. Future research could also explore the impact of speciality-specific interface adaptations, particularly in high-intensity environments such as intensive care or surgical settings^47^.

### Stewardship and safety (S)

Prescribing accuracy in simulation improved in association with the introduction of Ask Eolas^30,36^. No adverse events occurred during simulation^38,41^. These outcomes suggest that Ask Eolas may align prescribing behaviours with national antimicrobial resistance guidelines, thereby supporting broader antimicrobial stewardship objectives^35,36,41^. The inclusion of source citations adds a safety layer by spotlighting ambiguous or uncertain decisions^32,33^. As a policy priority, AI-CDSS certification processes should incorporate checks for alignment with stewardship protocols^36,41^. Longitudinal studies are warranted to assess the impact of such tools on prescribing behaviours across diverse clinical settings^44,46^. It is important to note the contrasting high environmental cost of training foundational LLMs with the relatively low energy consumption associated with Retrieval-Augmented Generation (RAG) inference on a pre-trained, smaller model, which is the architecture used. Further policy work will be needed to inform commissioning. This should accompany the ongoing system development guided by the PPI feedback, particularly focusing on mitigating potential prescribing biases and ensuring data governance compliance (anonymisation, security).

### Triage and confidence calibration (T)

The system would have benefited from a real-time confidence scoring framework that stratified outputs into three tiers^32,33^. This could have proceeded with recommendations, with scores of 80 or above being deemed safe to implement without further review; those scoring between 60 and 79 flagged for clinician discretion; and those below 60 referred for expert evaluation^33,36^. Participants using these scores could adjust their reliance on the tool, thereby minimising the risk of high-confidence but low-accuracy decisions^34,36^. This triaging mechanism functions not merely as a user feature but as a foundational safety and scaling strategy^33,36,38^. Regulatory guidelines should require AI-CDSS vendors to disclose their confidence calibration methods, and standardised thresholds should be developed across clinical domains to ensure consistency in triage practices^44,47^.

### Accountability and auditability (A)

All prescribing decisions generated by Ask Eolas were logged^38,41^. This approach enables robust auditability and error accountability for this service evaluation, both of which are essential for regulatory compliance and ongoing system improvement^44,46^. The availability of detailed logs facilitates continuous learning and enhances patient safety over time^38,41,46^. Regulatory bodies should require comprehensive logging of AI-CDSS outputs, including timestamps, override rationales, and supporting source documentation^45,47^. Simulation evaluations, such as those aligned with the SIROS framework, could serve as an effective exploration approach for adaptive systems seeking clinical development^44,46^.

### Implementation and interoperability (I)

The system was deployed via Microsoft Teams during the study, simulating real-world clinical workflows^30,33^. However, full-scale deployment challenges, including integration with live EHR systems, the risk of alert fatigue, and potential increases in documentation burden, were not comprehensively addressed in the current evaluation^35,39^. Despite encouraging outcomes in a controlled simulation environment, effective real-world implementation will require deep integration with clinical systems and adaptation to local workflows^39,45^. Policy support is needed for translational research that tests AI-CDSS tools across live, multi-site environments^44,47^. Additionally, shared evaluation metrics should be developed to enable consistent comparison of AI-CDSS tools across institutions, vendors, and patient populations^45^.

The randomised simulation design reduced selection bias and enabled a robust comparison of the Ask Eolas system against existing methods. The three-phase user testing framework progressively increased the clinical complexity of scenarios. This realistic approach assesses the system’s performance on both simple and nuanced simulation cases, while the feedback loop ensures the final evaluation is based on a refined product. The study also benefits from a diverse set of outcome measures. Beyond just prescribing accuracy, metrics include usability (SUS), cognitive workload (NASA-TLX), decision time, and user confidence. The adherence to standardised reporting frameworks like DECIDE-AI and SIROS^12,13^ enhances scientific rigour, and the active public and patient involvement (PPIE) ensures the system’s development is ethically informed and patient-centred. We acknowledge that clinical deviation is sometimes warranted for complex patients, but stress that (1) the simulated cases were non-complex, guideline-appropriate scenarios where adherence is expected; (2) the primary purpose of ‘Ask Eolas’ is fidelity to local guidelines, reducing errors due to misinterpretation; (3) the RAG-CDSS is intended as a Decision Support tool, not a ‘Decision Maker’. The 100% accuracy demonstrates the system’s ability to correctly retrieve and synthesise information, but the final decision remains with the prescribing clinician, allowing for appropriate deviation when clinically necessary.

A primary limitation is that this was a simulated evaluation without generalisable findings, and not a real-world implementation. The simulation environment may not fully capture the complexities and pressures of a busy clinical setting, which could lead to participants’ awareness of being studied influencing their behaviour. The small sample size of 45 participants undermines the generalisability of the findings and makes it difficult to perform robust stratified analyses by clinician role. The evaluation’s single-site nature means results may not be applicable to different healthcare settings. Lastly, the reliance on a pilot ‘bolt-on’ product of an app already in use could mean that the true impact might change if the system is fully integrated into existing clinical workflows. There would be merit in considering a ‘crossover’ design in the future, but the parallel-group design was necessary to avoid carry-over effects and contamination, which are highly likely in a simulation, learning-focused study. Our results may represent a ‘best-case’ scenario for adoption among highly motivated users.

This ‘Ask Eolas’ simulation evaluation demonstrates that AI-driven clinical decision support systems, when designed with clinician trust at their core, have the potential to meaningfully enhance antimicrobial prescribing accuracy, efficiency, and safety in a simulated context. This work encourages future work examining the deployment of large language model (LLM)-based Clinical Decision Support System tools in high-stakes clinical contexts, offering early evidence for their integration into real-world workflows. The study’s outcomes directly support the proposed TRUST-AI framework, which provides a structured policy and research agenda for the safe, ethical, and scalable deployment of this form of AI in healthcare. Through high-fidelity simulation, human-in-the-loop validation, and explainable architecture, ‘Ask Eolas’ establishes a simulated framework for future trials.

## Methods

### Study design

This study utilises a three-phase user testing framework with a randomised simulated evaluation design to evaluate the prescription accuracy of Ask Eolas, a RAG-enhanced CDSS for antimicrobial prescribing. The study follows the DECIDE-AI reporting guidelines and complies with the Standardised Iterative Reporting and Outcomes Simulation (SIROS) framework to ensure rigorous evaluation of early-phase AI systems^12,13^.

Forty-five healthcare professionals, invited by app user emails, were randomly allocated through computer-generated randomisation to one of three intervention groups, with fifteen participants per group. This utilised a think-aloud protocol of transcription using Microsoft Teams video conferencing software, with thematic analysis of the qualitative data using NVivo. The investigation employed a sequential three-phase design characterised by progressively increasing clinical complexity. Phase 1 encompassed low complexity scenarios involving straightforward antimicrobial prescribing cases with clear diagnostic criteria and standard treatment pathways. Phase 2 incorporated medium complexity cases requiring consideration of patient factors, comorbidities, and alternative treatment options. Phase 3 examined high-complexity scenarios involving multi-factorial cases with drug resistance patterns, multiple treatment failures, or rare infections necessitating specialist consultation. The details of each of the 45 cases are included as Supplementary Notes 4 in the Supplementary Information; these cases were authored in consultation with the infectious disease and antimicrobial stewardship leadership of the hospital, and were compliant with the hospital guidelines at the time of use. All three intervention groups underwent evaluation at each complexity phase to enable comparative assessment across the complete spectrum of clinical scenarios.

### Setting

**Fig. 1:**
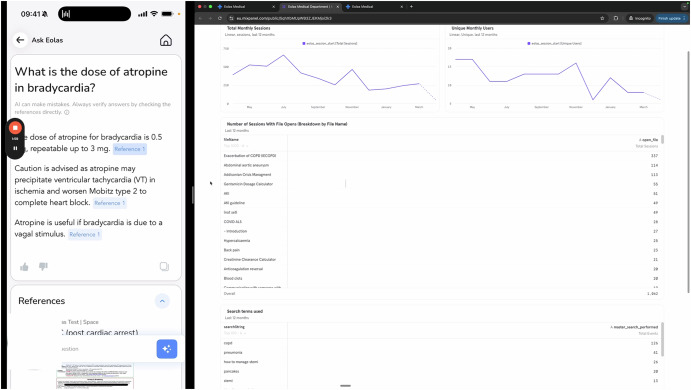
‘Ask Eolas’ interface screenshot (Credit Eolas Medical Ltd).

The evaluation was conducted using simulated cases modelled on real-world clinical scenarios within a large London teaching hospital environment, encompassing emergency departments, intensive care units, and speciality wards. Simulations representing the hospital’s prescribing pathways were delivered remotely through Microsoft Teams to model clinical documentation and prescribing workflows within a controlled virtual environment. The simulation environment was a standardised, non-patient-identifiable pathway ensuring all participants experienced the same layout and interface. In the Trust Guidelines group, participants accessed static PDF antimicrobial prescribing guidelines through the hospital’s intranet system, representing standard practice for guideline consultation within the clinical environment. In the Eolas App group, participants utilised the hospital-deployed mobile application providing structured access to antimicrobial guidelines with navigation organised by body system, reflecting the current digital service implemented within the hospital infrastructure. In the Ask Eolas group, participants engaged with an artificial intelligence-powered natural language interface integrated as an enhancement to the existing Eolas application. This RAG-enhanced CDSS incorporated natural language query processing for antimicrobial prescribing guidance, transparent outputs with clinical rationale and source citations, direct links to relevant hospital guideline sections, and recommendations anchored exclusively in the hospital’s antimicrobial prescribing guidelines. A structured “second-reader” workflow was implemented to minimise anchoring bias, whereby clinicians first formulated independent clinical decisions before artificial intelligence recommendations were presented. Figure 1 provides a screenshot of the Ask Eolas interface, showing both query and response, alongside the department analytics dashboard to support management in antimicrobial stewardship commissioning decisions.

### Recruitment and simulation procedure

Prescribers were recruited from clinical staff actively engaged in antimicrobial prescribing within the study institution. Recruitment targeted consultants, resident doctors, clinical pharmacists, and prescribing nurses, including advanced nurse practitioners. Inclusion criteria comprised active antimicrobial prescribing responsibilities, demonstrated familiarity with local antimicrobial guidelines, and current clinical practice at the study site. Exclusion criteria included prior exposure to the Ask Eolas system and inability to complete remote simulation tasks.

All simulations were conducted remotely by imitating the hospitals' prescribing pathways via Microsoft Teams with standardised protocols. Each participant completed informed consent procedures, received orientation to their assigned intervention, worked through clinical scenarios accurate to their designated study phase, utilised think-aloud protocols with concurrent verbalisation recorded via Microsoft Teams transcription, and participated in structured debriefing sessions between phases. Participants retained the right to withdraw from the study at any point during the investigation.

The researcher conducting the NASA-TLX and SUS scoring was a white male resident doctor in their late twenties; they were independent of the system development team and trained specifically on the standardised administration of NASA-TLX and SUS protocols.

### Primary outcomes

Prescribing accuracy was assessed through guideline adherence measured against internal hospital antimicrobial guidelines, evaluated across five domains: antibiotic selection accurate for infection type, correct route of administration, accurate dosage, suitable treatment duration, and consideration of local microbiology and resistance patterns. Given the aim of a CDSS to deliver perfect guideline fidelity, any failure in one of the five domains, regardless of the magnitude (e.g., 9 days instead of 10), resulted in the prescription being counted as inaccurate for the primary outcome; this was adjudicated according to the official Trust guidelines using a predefined mark scheme.

### Secondary outcomes

System usability was assessed using the validated 10-item System Usability Scale with scores ranging from 0 to 100. Cognitive workload was measured via the NASA Task Load Index, evaluating mental demand, physical demand, time pressure, effort required, perceived performance, and frustration level. Decision efficiency was captured through automated timestamp logs during simulation tasks. Clinical confidence was measured through self-reported confidence in prescribing decisions on a 0-100 visual analogue scale. User acceptance was evaluated through the likelihood to recommend the tool to colleagues using a 0–10 scale. Workflow integration was assessed through documentation of instances when clinician behaviour diverged from standard prescribing pathways. Qualitative insights were obtained through the think-aloud protocol data, exploring clinical reasoning processes, tooltip interpretation, and interface interactions. Source citations engagement was recorded through video logs. All user queries and the associated responses are captured as part of the retained audit logs from the video calls.

### Analytical framework

Performance metrics were stratified by clinician role, case complexity, and study phase to assess scalability across increasing clinical complexity. Statistical analysis employed Fisher’s exact test for the primary binary outcome, with statistical significance established at *p* < 0.05 and 95% confidence intervals reported where accurate. Fisher’s exact test was employed to analyse the primary binary outcome of prescribing accuracy due to the discrete nature of the data and relatively small sample sizes per intervention group (*n* = 15), providing exact p-values for comparing accurate prescription rates between the three groups. The number needed to treat (NNT) was calculated from the absolute risk reduction between intervention groups, with statistical significance established at *p* < 0.05.

Public contributors were actively involved in refining research questions, recruitment strategies, and outcome measures for the future exploration of similar applications of LLMs. In total, 150 members of the public expressed interest in taking part in our PPI session, with 10 selected to ensure a diverse group. Nine of these took part in a Microsoft Teams video call in February 2025 and were compensated in line with NIHR guidance and supported with preparatory materials and technical assistance. Participants were updated with the findings of the session. PPIE participants emphasised the need for transparency in AI decision-making, questioning data usage, reliability, and potential biases. AMR was a major concern, with calls for responsible prescribing to prevent misuse. Immunocompromised participants stressed the risks of increasing resistance. A patient-centred approach was advocated, ensuring AI tools are accessible, intuitive, and designed with patient input. Data security and AI bias were key issues, with concerns about underrepresented communities receiving inaccurate recommendations. Ethical AI development and diverse training data were seen as crucial. AI was viewed as an aid to clinical decision-making rather than a replacement for human judgement.

### Ethical considerations

The study received approval as a service evaluation from the organisation’s Audit Office (Ref: 1200). All data were anonymised in line with GDPR and NHS data security protocols. Informed consent was obtained, and participants could withdraw at any point. The suitability of this project for approval under the service evaluation pathway was confirmed by the host institution's authorising office and by using the online HRA decision support tool^48^. This Ask Eolas product does not receive any patient data, and so there is no confidentiality risk.

### Compliance with the SIROS framework^13^

Standardised evaluations were conducted to assess prescribing accuracy, the SUS, clinician confidence, and cognitive load. Data collection was rigorous, with comprehensive metrics logged to ensure accuracy. The design of the system was outcome-driven, aiming to improve antimicrobial prescribing, enhance usability, and reduce clinician workload. Finally, scalability testing ensured the system could handle a high volume of scenarios and diverse clinician input without performance degradation.

### Ethics statement

The study received approval as a service evaluation from the teaching hospital Audit Office (Ref: 1200). All data were anonymised in line with GDPR and NHS data security protocols. Informed consent was obtained, and participants could withdraw at any point. The suitability of this project for approval under the service evaluation pathway was confirmed with the hospital authorities using the HRA NHS ethics online tool^48^. No real patient data were used in simulations. All participants provided informed written consent.

## Supplementary information

Supplementary information

## Data Availability

Data is provided within the manuscript.
