# Supplementary material for: Enhancing quality of antimicrobial prescribing through ‘Ask Eolas’ (language model): a user-testing and simulation evaluation
Source: NPJ Antimicrob Resist. 2026 Mar 3;4:16. doi: 10.1038/s44259-026-00187-7 (PMC12957523; doi:10.1038/s44259-026-00187-7)
Supplement: Supplementary file 1 — Supplementary information [file 44259_2026_187_MOESM1_ESM.docx]

### **Supplementary Notes 1: Use of Generative AI in the Study**

**Generative AI Use Declaration:**

Yes – Generative AI (GAI) was used in this study, but solely as the subject of evaluation (Ask Eolas). It was not used to draft, edit, or revise the manuscript itself.

**Role of GAI in the Study:**

The system evaluated, *Ask Eolas*, is a clinical decision support system (CDSS) powered by a Retrieval-Augmented Generation (RAG) large language model. The AI model was integrated into a simulated prescribing environment to assess its usability, decision accuracy, and impact on clinician workflow in antimicrobial stewardship scenarios.

**Manuscript Authorship:**

All text in this manuscript was written and revised by human authors. No generative AI tools were employed during manuscript preparation. Authorship meets all ICMJE requirements.

**Technical and Ethical Compliance (Aligned with GAMER, DECIDE-AI, and SIROS):**

- **Tool Name and Model Type:** *Ask Eolas CDSS*, incorporating a transformer-based generative model augmented by a custom vector search index of institutional antimicrobial guidelines.
- **Customisation and Data Handling:** No fine-tuning was performed. All guideline data were embedded using sentence transformers and stored on secure, local NHS servers.
- **Prompting Strategy:** Prescribers entered free-text queries (e.g., “What is the recommended treatment for febrile neutropenia?”). The system returned natural-language responses grounded in local guidelines (of the teaching hospital.
- **Output Oversight:** A second-reader workflow was employed. Prescribers made initial decisions independently before reviewing AI outputs. Output safety and accuracy were evaluated by expert reviewers using a structured adjudication process.
- **Risk Mitigation Measures:** Outputs were accompanied by citations and rationale tooltips.
- **Data Privacy:** This study used simulated clinical scenarios only. No real patient data were processed. All simulation data were stored and analysed on secure NHS infrastructure.
- **Public and Patient Involvement:** AI transparency, fairness, and usability features were co-designed with public contributors. No patient-identifiable data were used at any point.

**Supplementary Notes 2: PPIE NVivo Code Examples (February 2025)**

Guest 1:

Text Segment: "I've had various courses of antibiotics in the past. Prone to chest infections... I don't have an issue with AI, but I think confidentiality is important."

Parent Node: Participant Demographics, AI in Antimicrobial Prescribing

Codes: Experience (Antimicrobial Treatment), Trust (AI)

Justification: The participant describes personal experience with antimicrobial treatment and expresses moderate trust in AI but highlights confidentiality concerns.

Guest 3:

Text Segment: "My father contracted C. diff in hospital, and my mother had pleurisy, requiring multiple antibiotic courses."

Parent Node: Patient-Centred Design, Perspectives on AMR

Codes: Patient Voice, AMR Concern

Justification: The participant shares personal caregiving experience highlighting AMR risks and patient involvement in treatment decisions.

Guest 4:

Text Segment: "I’m immunocompromised and constantly on antibiotics. I’m concerned about data safety and confidentiality."

Parent Node: Participant Demographics, Ethics and Safety

Codes: Experience (Antimicrobial Treatment), Privacy

Justification: The participant provides personal medical history and raises privacy concerns regarding AI and data usage.

Guest 5:

Text Segment: "AI models should be trained on diverse datasets to prevent bias in decision-making."

Parent Node: Ethics and Safety, AI in Antimicrobial Prescribing

Codes: Fairness, AI Limitations

Justification: The participant identifies a key limitation of AI—potential biases due to incomplete data representation.

Guest 6:

Text Segment: "Will AI be able to consider multiple comorbidities?"

Parent Node: AI in Antimicrobial Prescribing, Impact on Clinical Decision-Making

Codes: AI Limitations, Efficiency

Justification: Raises concerns about AI's capability in handling complex health conditions and improving efficiency.

**Supplementary Notes 3 NVivo Coding Framework for simulation cases analysis**

**Parent Nodes and Sample Child Nodes:**

- **Decision-Making Process**
  - Clinical reasoning
  - Guideline access methods
  - Use of scoring tools
- **Barriers and Facilitators**
  - Format issues (PDF/App/AI)
  - Search/navigation challenges
  - Speed/reliability
- **Usability Feedback**
  - Interface layout
  - Learnability
  - Mobile performance
- **Trust and Transparency**
  - Source verification
  - Confidence in AI
  - Output reliability
- **Suggested Improvements**
  - Structured prompts
  - Visual summaries
  - Customisation options
- **Emotional Responses**
  - Reassurance
  - Frustration
  - Confidence
  - Anxiety

**Supplementary Notes 4: Prescribing Cases Details (45 Cases)**

All cases are compliant with host hospital Trust antimicrobial guidelines, stewardship, and documentation standards.

## Case Classification System

### Level 1 (Basic) – 15 cases

- Simple infections
- No significant comorbidities
- Standard treatment durations
- Basic monitoring required
- No specialist input needed

### Level 2 (Intermediate) - 15 cases

- Moderate complexity
- Some comorbidities
- Extended treatment durations
- Regular monitoring required
- May need specialist input

### Level 3 (Complex) - 15 cases

- High complexity
- Multiple comorbidities
- Complex treatment regimens
- Intensive monitoring required
- Specialist input essential

## Level 1 Cases (Basic)

### Summary Table: Level 1 Cases 1–15

| **Case #** | **Case ID** | **Infection Type** | **Key Features** |
| --- | --- | --- | --- |
| 1 | RESP-B001 | Acute Bronchitis | No comorbidities, viral |
| 2 | RESP-B002 | CAP | CURB-65=1, no comorbidities |
| 3 | RESP-B003 | COPD Exacerbation | COPD, no severe features |
| 4 | RESP-B004 | Sinusitis | No comorbidities, mild |
| 5 | UTI-B001 | Simple UTI (Female) | No comorbidities, simple |
| 6 | UTI-B002 | Simple UTI (Male) | No comorbidities, simple |
| 7 | UTI-B003 | Asymptomatic Bacteriuria | No symptoms, elderly |
| 8 | UTI-B004 | Simple Cystitis | No comorbidities, simple |
| 9 | SKIN-B001 | Simple Cellulitis | No comorbidities, simple |
| 10 | SKIN-B002 | Simple Abscess | No comorbidities, simple |
| 11 | SKIN-B003 | Simple Impetigo | Paediatric, localised |
| 12 | SKIN-B004 | Simple Wound Infection | No comorbidities, simple |
| 13 | ENT-B001 | Acute Otitis Media | Paediatric, simple |
| 14 | ENT-B002 | Acute Pharyngitis | No comorbidities, simple |
| 15 | ENT-B003 | Simple Tonsillitis | No comorbidities, viral |

### Case 1: Simple Acute Bronchitis

Case ID: RESP-B001

#### Clinical Scenario

- 35-year-old male
- 3-day history of cough, sputum
- Past medical history: None
- Observations: Temp 37.2°C, BP 120/75, HR 80
- Chest clear, no focal signs
- No allergies
- eGFR: 90 ml/min
- Not immunosuppressed

### Case 2: Community-Acquired Pneumonia

Case ID: RESP-B002

#### Clinical Scenario

- 45-year-old male
- 3-day history of cough, fever, breathlessness
- Past medical history: None
- Observations: Temp 38.2°C, BP 130/80, HR 95, RR 22, SpO2 95%
- Right lower lobe crackles
- No allergies
- eGFR: 90 ml/min
- Not immunosuppressed
- CURB-65 score: 1

### Case 3: Acute Exacerbation of COPD

Case ID: RESP-B003

#### Clinical Scenario

- 60-year-old male
- 2-day history of increased sputum, breathlessness
- Past medical history: COPD
- Observations: Temp 37.5°C, BP 135/85, HR 90
- Widespread wheeze, no focal signs
- No allergies
- eGFR: 75 ml/min
- Not immunosuppressed

### Case 4: Simple Sinusitis

Case ID: RESP-B004

#### Clinical Scenario

- 30-year-old female
- 7-day history of facial pain, nasal discharge
- Past medical history: None
- Observations: Temp 37.0°C, BP 120/75, HR 80
- Tenderness over maxillary sinuses
- No allergies
- eGFR: 90 ml/min
- Not immunosuppressed

### Case 5: Simple UTI in Young Female

Case ID: UTI-B001

#### Clinical Scenario

- 25-year-old female
- 2-day history of dysuria, frequency
- Past medical history: None
- Observations: Temp 37.0°C, BP 120/75, HR 80
- Suprapubic tenderness
- No allergies
- eGFR: 90 ml/min
- Not immunosuppressed

### Case 6: Simple UTI in Young Male

Case ID: UTI-B002

#### Clinical Scenario

- 30-year-old male
- 2-day history of dysuria, frequency
- Past medical history: None
- Observations: Temp 37.0°C, BP 125/80, HR 80
- Suprapubic tenderness
- No allergies
- eGFR: 90 ml/min
- Not immunosuppressed

### Case 7: Asymptomatic Bacteriuria

Case ID: UTI-B003

#### Clinical Scenario

- 70-year-old female
- No urinary symptoms
- Past medical history: None
- Observations: Temp 36.8°C, BP 130/80, HR 75
- No urinary symptoms
- No allergies
- eGFR: 60 ml/min
- Not immunosuppressed
- Urine dip: Leucocytes +, Nitrites +

### Case 8: Simple Cystitis

Case ID: UTI-B004

#### Clinical Scenario

- 40-year-old female
- 1-day history of dysuria, frequency
- Past medical history: None
- Observations: Temp 37.0°C, BP 120/75, HR 80
- Suprapubic tenderness
- No allergies
- eGFR: 85 ml/min
- Not immunosuppressed

### Case 9: Simple Cellulitis

Case ID: SKIN-B001

#### Clinical Scenario

- 45-year-old male
- 2-day history of leg redness, pain
- Past medical history: None
- Observations: Temp 37.5°C, BP 130/80, HR 85
- 5cm area of erythema, warm, tender
- No allergies
- eGFR: 90 ml/min
- Not immunosuppressed

### Case 10: Simple Abscess

Case ID: SKIN-B002

#### Clinical Scenario

- 35-year-old female
- 3-day history of painful lump
- Past medical history: None
- Observations: Temp 37.0°C, BP 120/75, HR 80
- 3cm fluctuant abscess, no surrounding cellulitis
- No allergies
- eGFR: 90 ml/min
- Not immunosuppressed

### Case 11: Simple Impetigo

Case ID: SKIN-B003

#### Clinical Scenario

- 5-year-old male
- 2-day history of facial rash
- Past medical history: None
- Observations: Temp 36.8°C, BP 90/60, HR 100
- Multiple small, crusted lesions
- No allergies
- Normal renal function
- Not immunosuppressed

### Case 12: Simple Wound Infection

Case ID: SKIN-B004

#### Clinical Scenario

- 40-year-old female
- 2-day history of wound pain, redness
- Past medical history: None
- Observations: Temp 37.0°C, BP 120/75, HR 80
- 3cm surgical wound, mild erythema
- No allergies
- eGFR: 90 ml/min
- Not immunosuppressed

### Case 13: Acute Otitis Media

Case ID: ENT-B001

#### Clinical Scenario

- 3-year-old female
- 2-day history of ear pain, fever
- Past medical history: None
- Observations: Temp 38.0°C, BP 90/60, HR 110
- Red, bulging tympanic membrane
- No allergies
- Normal renal function
- Not immunosuppressed

### Case 14: Acute Pharyngitis

Case ID: ENT-B002

#### Clinical Scenario

- 20-year-old male
- 3-day history of sore throat, fever
- Past medical history: None
- Observations: Temp 38.8°C, BP 100/75, HR 125
- Tonsillar exudate, tender nodes
- No allergies
- eGFR: 90 ml/min
- Not immunosuppressed

### Case 15: Simple Tonsillitis

Case ID: ENT-B003

#### Clinical Scenario

- 25-year-old female
- 3-day history of sore throat, difficulty swallowing
- Past medical history: None
- Observations: Temp 37.5°C, BP 120/75, HR 85
- Enlarged tonsils, no exudate
- No allergies
- eGFR: 90 ml/min
- Not immunosuppressed

## Level 2 Cases (Intermediate)

### Summary Table: Level 2 Cases 1–15

| **Case #** | **Case ID** | **Infection Type** | **Key Features** |
| --- | --- | --- | --- |
| 1 | RESP-I001 | CAP with COPD | COPD, moderate severity |
| 2 | RESP-I002 | Bronchiectasis Exacerbation | Previous Pseudomonas |
| 3 | HAP-I001 | Hospital-Acquired Pneumonia | Hospital stay, moderate |
| 4 | COPD-I001 | COPD with MRSA | COPD, previous MRSA |
| 5 | UTI-I001 | Complicated UTI | Renal stones, simple |
| 6 | UTI-I002 | UTI with Catheter | Indwelling catheter |
| 7 | SKIN-I001 | Cellulitis with Diabetes | Diabetes, simple |
| 8 | SKIN-I002 | Diabetic Foot Infection | Diabetes, foot infection |
| 9 | AMR-I001 | ESBL UTI | ESBL-producing organism |
| 10 | AMR-I002 | MRSA Bacteremia | MRSA bloodstream infection |
| 11 | RESP-I003 | CAP with Heart Failure | Heart failure, moderate |
| 12 | UTI-I003 | UTI with Renal Impairment | Renal impairment, simple |
| 13 | SKIN-I003 | Cellulitis with Obesity | Obesity, simple |
| 14 | ENT-I001 | Sinusitis with Asthma | Asthma, moderate severity |
| 15 | EYE-I001 | Endophthalmitis | Eye infection, moderate |

### Case 1: Community-Acquired Pneumonia with COPD

Case ID: RESP-I001

#### Clinical Scenario

- 65-year-old male
- 3-day history of cough, fever, breathlessness
- Past medical history: COPD (FEV1 40%), IHD
- Observations: Temp 38.5°C, BP 140/85, HR 95, RR 24, SpO2 92%
- Bilateral crackles, reduced air entry
- No allergies
- eGFR: 60 ml/min
- Not immunosuppressed
- CURB-65 score: 2

### Case 2: Bronchiectasis Exacerbation

Case ID: RESP-I002

#### Clinical Scenario

- 55-year-old male
- 2-day history of increased sputum, breathlessness
- Past medical history: COPD, bronchiectasis
- Observations: Temp 38.0°C, BP 130/80, HR 90
- Widespread wheeze, reduced air entry
- No allergies
- eGFR: 70 ml/min
- Not immunosuppressed

### Case 3: Hospital-Acquired Pneumonia

Case ID: HAP-I001

#### Clinical Scenario

- 75-year-old male
- 2-day history of fever, cough, breathlessness
- Past medical history: COPD, IHD
- Observations: Temp 38.5°C, BP 140/85, HR 95, RR 24, SpO2 92%
- Bilateral crackles, reduced air entry
- No allergies
- eGFR: 60 ml/min
- Not immunosuppressed

### Case 4: COPD with MRSA

Case ID: COPD-I001

#### Clinical Scenario

- 60-year-old male
- 2-day history of increased sputum, breathlessness
- Past medical history: COPD, previous MRSA
- Observations: Temp 38.0°C, BP 130/80, HR 90
- Widespread wheeze, reduced air entry
- No allergies
- eGFR: 70 ml/min
- Not immunosuppressed

### Case 5: Complicated UTI

Case ID: UTI-I001

#### Clinical Scenario

- 50-year-old female
- 2-day history of dysuria, frequency
- Past medical history: Renal stones, no previous UTIs
- Observations: Temp 37.5°C, BP 120/75, HR 80
- Suprapubic tenderness
- No allergies
- eGFR: 90 ml/min
- Not immunosuppressed

### Case 6: UTI with Catheter

Case ID: UTI-I002

#### Clinical Scenario

- 70-year-old female
- 2-day history of dysuria, frequency
- Past medical history: Indwelling catheter
- Observations: Temp 37.5°C, BP 120/75, HR 80
- Suprapubic tenderness
- No allergies
- eGFR: 90 ml/min
- Not immunosuppressed

### Case 7: Cellulitis with Diabetes

Case ID: SKIN-I001

#### Clinical Scenario

- 55-year-old female
- 2-day history of leg redness, pain
- Past medical history: Diabetes
- Observations: Temp 37.5°C, BP 120/75, HR 80
- 5cm area of erythema, warm, tender
- No allergies
- eGFR: 90 ml/min
- Not immunosuppressed

### Case 8: Diabetic Foot Infection

Case ID: SKIN-I002

#### Clinical Scenario

- 60-year-old female
- 2-day history of foot pain, redness
- Past medical history: Type 2 Diabetes Mellitus
- Observations: Temp 37.5°C, BP 120/75, HR 80
- 3cm area of erythema, warm, tender
- No allergies
- eGFR: 90 ml/min
- Not immunosuppressed

### Case 9: ESBL UTI

Case ID: AMR-I001

#### Clinical Scenario

- 45-year-old male
- 2-day history of dysuria, fever
- Past medical history: ESBL-producing organism
- Observations: Temp 37.5°C, BP 120/75, HR 80
- Suprapubic tenderness
- No allergies
- eGFR: 90 ml/min
- Not immunosuppressed

### Case 10: MRSA Bacteraemia

Case ID: AMR-I002

#### Clinical Scenario

- 50-year-old female
- 2-day history of fever, chills
- Past medical history: MRSA bloodstream infection
- Observations: Temp 39.5°C, BP 120/75, HR 123
- No systemic features
- No allergies
- eGFR: 90 ml/min
- Not immunosuppressed

### Case 11: CAP with Heart Failure

Case ID: RESP-I003

#### Clinical Scenario

- 65-year-old male
- 3-day history of cough, fever, breathlessness
- Past medical history: COPD (FEV1 40%), IHD, CKD stage 3, Type 2 Diabetes Mellitus
- Observations: Temp 38.5°C, BP 140/85, HR 95, RR 24, SpO2 92%
- Bilateral crackles, reduced air entry
- No allergies
- eGFR: 35 ml/min
- Not immunosuppressed
- CURB-65 score: 4

### Case 12: UTI with Renal Impairment

Case ID: UTI-I003

#### Clinical Scenario

- 50-year-old female
- 2-day history of dysuria, frequency
- Past medical history: CKD stage 4
- Observations: Temp 37.5°C, BP 130/80, HR 85
- Urine dipstick: Leucocytes +, Nitrites +
- No allergies
- eGFR: 25 ml/min
- Not immunosuppressed
- CURB-65 score: 2

### Case 13: Cellulitis with Obesity

Case ID: SKIN-I003

#### Clinical Scenario

- 55-year-old female
- 2-day history of leg redness, pain
- Past medical history: Obesity
- Observations: Temp 37.5°C, BP 120/75, HR 80
- 5cm area of erythema, warm, tender
- No allergies
- eGFR: 90 ml/min
- Not immunosuppressed

### Case 14: Sinusitis with Asthma

Case ID: ENT-I001

#### Clinical Scenario

- 30-year-old male
- 5-day history of facial pain, nasal congestion
- Past medical history: Asthma, COPD
- Observations: Temp 37.5°C, BP 120/75, HR 85
- Facial tenderness, nasal discharge
- No allergies
- eGFR: 90 ml/min
- Not immunosuppressed

### Case 15: Endophthalmitis

Case ID: EYE-I001

#### Clinical Scenario

- 40-year-old male
- 1-day history of eye pain, vision loss
- Past medical history: Diabetes, recent cataract surgery
- Observations: Temp 37.5°C, BP 140/85, HR 85
- Eye inflammation, reduced vision
- No allergies
- eGFR: 70 ml/min
- Not immunosuppressed

## Level 3 Cases (Complex)

### Summary Table: Level 3 Cases 1–15

| **Case #** | **Case ID** | **Infection Type** | **Key Features** |
| --- | --- | --- | --- |
| 1 | RESP-C001 | Severe CAP with Multiple Comorbidities | Multiple comorbidities, severe |
| 2 | RESP-C002 | VAP with Multi-resistant Organisms | VAP, multi-resistant |
| 3 | RESP-C003 | CAP with Septic Shock | Septic shock, severe |
| 4 | UTI-C001 | UTI with Sepsis | Urosepsis, severe |
| 5 | SKIN-C001 | Necrotising Fasciitis | Necrotising infection, severe |
| 6 | SKIN-C002 | Fournier's Gangrene | Fournier's gangrene, severe |
| 7 | CNS-C001 | Brain Abscess | Brain abscess, severe |
| 8 | BONE-C001 | Osteomyelitis with MRSA | Osteomyelitis, MRSA, severe |
| 9 | RESP-C004 | CAP with ARDS | ARDS, severe respiratory |
| 10 | RESP-C005 | VAP with Pseudomonas | Pseudomonas, severe |
| 11 | UTI-C002 | UTI with Renal Failure | Renal failure, severe |
| 12 | GI-C001 | Diverticulitis with Peritonitis | Peritonitis, severe |
| 13 | BONE-C002 | Osteomyelitis with Diabetes | Diabetes, severe |
| 14 | CNS-C002 | Meningitis with Sepsis | Sepsis, severe |
| 15 | RESP-C006 | CAP with Heart Failure | Heart failure, severe |

### Case 1: Severe CAP with Multiple Comorbidities

Case ID: RESP-C001

#### Clinical Scenario

- 75-year-old male
- 3-day history of cough, fever, breathlessness
- Past medical history: COPD (FEV1 30%), IHD, CKD stage 3, Diabetes
- Observations: Temp 39.0°C, BP 140/85, HR 110, RR 28, SpO2 88%
- Bilateral crackles, reduced air entry
- No allergies
- eGFR: 35 ml/min
- Not immunosuppressed
- CURB-65 score: 4

### Case 2: VAP with Multi-resistant Organisms

Case ID: RESP-C002

#### Clinical Scenario

- 65-year-old male
- 5-day history of fever, cough, breathlessness
- Past medical history: COPD, recent intubation currently on ventilator
- Observations: Temp 38.5°C, BP 140/85, HR 100, RR 25, SpO2 90%
- Bilateral crackles, reduced air entry
- No allergies
- eGFR: 60 ml/min
- Not immunosuppressed

### Case 3: CAP with Septic Shock

Case ID: RESP-C003

#### Clinical Scenario

- 70-year-old male
- 2-day history of fever, cough, confusion
- Past medical history: COPD, IHD
- Observations: Temp 39.5°C, BP 85/50, HR 120, RR 30, SpO2 85%
- Bilateral crackles, reduced air entry
- No allergies
- eGFR: 50 ml/min
- Not immunosuppressed

### Case 4: UTI with Sepsis

Case ID: UTI-C001

#### Clinical Scenario

- 75-year-old female
- 1-day history of fever, confusion, dysuria
- Past medical history: Type 2 Diabetes Mellitus, CKD stage 3
- Observations: Temp 39.0°C, BP 90/60, HR 110, RR 24, SpO2 92%
- Suprapubic tenderness
- No allergies
- eGFR: 35 ml/min
- Not immunosuppressed

### Case 5: Necrotising Fasciitis

Case ID: SKIN-C001

#### Clinical Scenario

- 60-year-old male
- 1-day history of severe leg pain, swelling
- Past medical history: Diabetes, obesity
- Observations: Temp 39.5°C, BP 95/65, HR 115, RR 26, SpO2 90%
- Severe leg swelling, crepitus, pain out of proportion
- No allergies
- eGFR: 60 ml/min
- Not immunosuppressed

### Case 6: Fournier's Gangrene

Case ID: SKIN-C002

#### Clinical Scenario

- 65-year-old male
- 1-day history of severe perineal pain, swelling
- Past medical history: Diabetes, obesity
- Observations: Temp 39.0°C, BP 90/60, HR 110, RR 24, SpO2 92%
- Perineal swelling, crepitus, severe pain
- No allergies
- eGFR: 50 ml/min
- Not immunosuppressed

### Case 7: Brain Abscess

Case ID: CNS-C001

#### Clinical Scenario

- 45-year-old male
- 3-day history of headache, confusion, fever
- Past medical history: Recent dental work
- Observations: Temp 38.5°C, BP 140/90, HR 95, RR 20, SpO2 95%
- Focal neurological signs
- No allergies
- eGFR: 80 ml/min
- Not immunosuppressed

### Case 8: Osteomyelitis with MRSA

Case ID: BONE-C001

#### Clinical Scenario

- 55-year-old male
- 2-week history of leg pain, swelling
- Past medical history: Type 2 Diabetes Mellitus, previous MRSA
- Observations: Temp 38.0°C, BP 130/80, HR 90, RR 20, SpO2 95%
- Leg swelling, tenderness, limited movement
- No allergies
- eGFR: 70 ml/min
- Not immunosuppressed

### Case 9: CAP with ARDS

Case ID: RESP-C004

#### Clinical Scenario

- 70-year-old male
- 2-day history of fever, cough, severe breathlessness
- Past medical history: COPD, IHD
- Observations: Temp 39.0°C, BP 100/70, HR 110, RR 35, SpO2 80%
- Bilateral crackles, reduced air entry
- No allergies
- eGFR: 60 ml/min
- Not immunosuppressed

### Case 10: VAP with Pseudomonas

Case ID: RESP-C005

#### Clinical Scenario

- 65-year-old male
- 5-day history of fever, cough, breathlessness
- Past medical history: COPD, recent intubation
- Observations: Temp 38.5°C, BP 140/85, HR 100, RR 25, SpO2 90%
- Bilateral crackles, reduced air entry
- No allergies
- eGFR: 60 ml/min
- Not immunosuppressed

### Case 11: UTI with Renal Failure

Case ID: UTI-C002

#### Clinical Scenario

- 75-year-old female
- 1-day history of fever, confusion, dysuria
- Past medical history: CKD stage 5, diabetes
- Observations: Temp 39.0°C, BP 90/60, HR 110, RR 24, SpO2 92%
- Suprapubic tenderness
- No allergies
- eGFR: 15 ml/min
- Not immunosuppressed

### Case 12: Diverticulitis with Peritonitis

Case ID: GI-C001

#### Clinical Scenario

- 70-year-old male
- 1-day history of severe left lower abdominal pain, fever
- Past medical history: Diverticular disease, obesity
- Observations: Temp 39.0°C, BP 90/60, HR 110, RR 24, SpO2 92%
- Left lower quadrant tenderness, rebound tenderness
- No allergies
- eGFR: 60 ml/min
- Not immunosuppressed

### Case 13: Osteomyelitis with Diabetes

Case ID: BONE-C002

#### Clinical Scenario

- 65-year-old male
- 2-week history of foot pain, swelling, fever
- Past medical history: Type 2 Diabetes Mellitus, peripheral neuropathy
- Observations: Temp 38.5°C, BP 130/80, HR 95, RR 22, SpO2 95%
- Foot swelling, tenderness, reduced sensation
- No allergies
- eGFR: 60 ml/min
- Not immunosuppressed

### Case 14: Meningitis with Sepsis

Case ID: CNS-C002

#### Clinical Scenario

- 45-year-old male
- 1-day history of severe headache, fever, altered mental status
- Past medical history: Recent sinusitis
- Observations: Temp 39.5°C, BP 85/55, HR 120, RR 28, SpO2 88%
- Neck stiffness, photophobia, focal neurological signs
- No allergies
- eGFR: 80 ml/min
- Not immunosuppressed

### Case 15: CAP with Heart Failure

Case ID: RESP-C006

#### Clinical Scenario

- 75-year-old male
- 2-day history of cough, fever, severe breathlessness
- Past medical history: Heart failure (EF 25%), COPD, diabetes
- Observations: Temp 38.5°C, BP 100/70, HR 110, RR 30, SpO2 85%
- Bilateral crackles, reduced air entry, peripheral edema
- No allergies and not immunosuppressed
- eGFR: 40 ml/min
